# Muscle Fatigue Revisited – Insights From Optically Pumped Magnetometers

**DOI:** 10.3389/fphys.2021.724755

**Published:** 2021-12-17

**Authors:** Davide Sometti, Lorenzo Semeia, Sangyeob Baek, Hui Chen, Giulia Righetti, Juergen Dax, Cornelius Kronlage, Milena Kirchgässner, Alyssa Romano, Johanna Heilos, Deborah Staber, Julia Oppold, Thomas Middelmann, Christoph Braun, Philip Broser, Justus Marquetand

**Affiliations:** ^1^Department of Neural Dynamics and Magnetoencephalography, Hertie-Institute for Clinical Brain Research, University of Tübingen, Tübingen, Germany; ^2^MEG-Center, University of Tübingen, Tübingen, Germany; ^3^Graduate Training Centre of Neuroscience, International Max Planck Research School, University of Tübingen, Tübingen, Germany; ^4^Center for Pediatric Clinical Studies, University of Tübingen, Tübingen, Germany; ^5^German Center for Diabetes Research (DZD), IDM/fMEG Center of the Helmholtz Center Munich at the University of Tübingen, University of Tübingen, Tübingen, Germany; ^6^Center for Ophthalmology, University of Tübingen, Tübingen, Germany; ^7^Department of Epileptology, Hertie-Institute for Clinical Brain Research, University of Tübingen, Tübingen, Germany; ^8^Department of Biosignals, Physikalisch-Technische Bundesanstalt (PTB), Berlin, Germany; ^9^Center for Mind/Brain Sciences (CIMeC), University of Trento, Rovereto, Italy; ^10^Department of Psychology and Cognitive Science (DiPsCo), University of Trento, Rovereto, Italy; ^11^Children’s Hospital of Eastern Switzerland, Sankt Gallen, Switzerland

**Keywords:** OPM, sEMG, magnetomyography, muscle fatigue, quantum sensors

## Abstract

So far, surface electromyography (sEMG) has been the method of choice to detect and evaluate muscle fatigue. However, recent advancements in non-cryogenic quantum sensors, such as optically pumped magnetometers (OPMs), enable interesting possibilities to flexibly record biomagnetic signals. Yet, a magnetomyographic investigation of muscular fatigue is still missing. Here, we simultaneously used sEMG (4 surface electrode) and OPM-based magnetomyography (OPM-MMG, 4 sensors) to detect muscle fatigue during a 3 × 1-min isometric contractions of the left rectus femoris muscle in 7 healthy participants. Both signals exhibited the characteristic spectral compression distinctive for muscle fatigue. OPM-MMG and sEMG slope values, used to quantify the spectral compression of the signals, were positively correlated, displaying similarity between the techniques. Additionally, the analysis of the different components of the magnetic field vector enabled speculations regarding the propagation of the muscle action potentials (MAPs). Altogether these results show the feasibility of the magnetomyographic approach with OPMs and propose a potential alternative to sEMG for the study of muscle fatigue.

## Introduction

Muscle fatigue is defined as a decrease in maximal force production in response to sustained or repetitive muscular activity (Enoka and Duchateau, 2008; Wan et al., 2017). The mechanisms underlying this process are manifold. One major contributor seems to be the accumulation of lactate in the muscles as a byproduct of anaerobic glycolysis during condition of high energy demand (Tesch et al., 1978; Sahlin, 1986; Ishii and Nishida, 2013); however, there is no consensus and existing evidence questions this assumption (Brooks, 2001; Gladden, 2004; Sola-Penna, 2008). Therefore, a global mechanism cannot be addressed (Allen et al., 2008), leading to a variety of available protocols and methods for the study of muscular fatigue (Vøllestad, 1997; Cifrek et al., 2009; Ma et al., 2009; Muramatsu and Kobayashi, 2014).

Surface electromyography (sEMG) is a commonly used and well-established means to continuously and non-invasively monitor muscle fatigue (Jurell, 1998; Cifrek et al., 2009; Marco et al., 2017; Liu et al., 2019) by recording the muscle action potentials (MAP) produced during muscle contraction. An early investigation of the frequency components of the electromyography (EMG) signal reported a shift of the power spectrum toward lower frequencies as an index of muscle fatigue (Kogi and Hakamada, 1962). A phenomenon also referred to as spectral compression (De Luca, 1993; Lowery et al., 2000). This result has been replicated several times (Viitasalo and Komi, 1977; Petrofsky et al., 1982; De Luca, 1984; Duchêne and Goubel, 1990; Doud and Walsh, 1995; Lowery et al., 2000; Beck et al., 2014) confirming the validity of this measure for the evaluation of muscle fatigue (Cifrek et al., 2009). Nevertheless, in spite of the recognized reliability of sEMG, the last few decades have seen the progressive development and miniaturization of magnetic sensors (Zuo et al., 2020), for the study of muscle activity.

Magnetomyography (MMG), as defined in the pioneering work by Cohen and Givler (1972), seeks to investigate muscle contraction physiology by recording the magnetic fields which arise from the same ionic current that generate the EMG signal (Cohen and Givler, 1972; Parker and Wikswo, 1997). The working principle of MMG is based on Biot-Savart law. The ionic current flowing within a muscle fiber generates concentric and circular magnetic fields, which can be recorded with a sufficiently sensitive magnetometer. Even though MMG and EMG signals originate from the same ionic currents, and present comparable temporal and spectral profile (Cohen and Givler, 1972; Parker and Wikswo, 1997), the potential superiority of MMG for the investigation of muscle contractions has been suggested (Garcia and Baffa, 2015). The magnetic permeability of human tissue is almost the same as that of empty space (Schenck, 1996); therefore, MMG signals are much less distorted by the different tissue layer interposed between the source and the skin surface. Besides, noise voltages originating at the electrode-skin interface during sEMG recording might interfere with the signal (Huigen et al., 2002; De Luca et al., 2010) and could be avoided using contactless MMG recording. Indeed, to date, MMG has shown a broad variety of applications (Fenici et al., 2005; Broser et al., 2018; Escalona-Vargas et al., 2019; Strand et al., 2019; Barchiesi et al., 2020; Blanks and Eswaran, 2020; Elzenheimer et al., 2020; Llinás et al., 2020; Marquetand et al., 2021; Broser et al., 2021a,b).

Here, we use MMG with Optically Pumped Magnetometers (OPMs) to monitor muscle fatigue of the rectus femoris muscle during an isometric contraction. The small size of the sensors and the unnecessity of cryogenic cooling (for a detailed technical description of OPM sensors see: Shah and Wakai, 2013; Boto et al., 2017; Tierney et al., 2019) allow a simultaneous Optically Pumped Magnetometer Magnetomyography (OPM-MMG) and sEMG recording. We compare the spectral profile of the signals over time using time-frequency decomposition and quantify the spectral compression by calculating the decrease of the spectral center of gravity over time. Additionally, we correlate OPM-MMG and sEMG slope values, used to quantify the spectral compression of the signals, to estimate the similarity between the techniques. Finally, we analyze the signal magnitude of the different components of the magnetic field vector to infer coarse spatial information regarding the main direction of propagation of the MAPs during isometric contraction.

## Materials and Methods

### Participants

Twelve healthy subjects participated in the study (6 males; mean age: 26.5 ± 2.9 SD years; and mean body-mass-index: 22.3 ± 1.5 SD kg/m^2^). The experiment was conducted at the MEG Center of the University of Tübingen (Germany) in March 2021, in accordance with the standards of the World Medical Association. All the participants are also authors of this publication and gave informed consent for their data to be published.

### Experimental Setup

Prior to the measurement, ultrasound imaging (Mindray TE7, 14Mhz-linear probe) of the left rectus femoris muscle was performed for each subject by a certified ultrasound user (JM) to determine the longitudinal axis of the muscle. Following, participants sat down on a comfortable chair inside a magnetically shielded room (Ak3b, VAC Vacuumschmelze, Hanau, Germany), extending the legs on the chair at a controlled knee angle of 150°.

**Figure 1:**
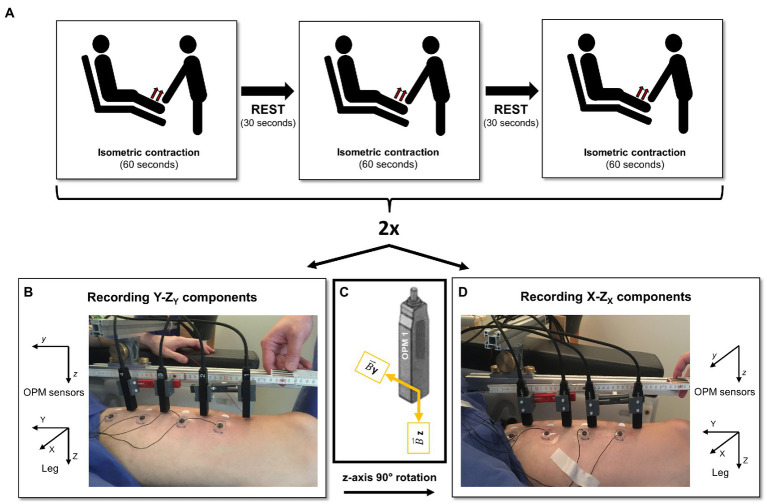
Experimental setup. **(A)** Experimental design. **(B)** Experimental setup during the recording of the Y and Z_Y_ components, with reference to the reported leg coordinates system. **(C)** The utilized optically pumped magnetometer (OPM) sensors could record only two components per time (*y* and *z*); therefore, they were rotated by 90° around the z-axis to also record the signal of the X component. **(D)** Experimental setup during the recording of the X and Z_X_ spatial components, referred to the reported leg coordinates system. Note here the different orientation of the sensors.

Four OPM sensors (QZFM-gen-1.5, QuSpin Inc., Louisville, CO, United States) were placed linearly in a distal to proximal order parallel to the longitudinal extent of the left rectus femoris muscle. The OPMs were held by a custom-built plastic holder attached to an aluminum structure. The sensors were placed at 6 cm distance from each other, with the first sensor placed 6 cm proximal to the patella, and 1–3 cm (mean distance 2.03 ± 0.65 SD cm) above the skin surface. In an alternating pattern, with respect to the OPM sensors, 4 paramagnetic surface electrodes (Conmed, Cleartrace^2^ MR-ECG-electrodes) were place along the rectus femoris muscle for the recording of the electromyographic signal (Figures 1B,D). Additionally, one ground and one reference electrode were placed, respectively, on the right shoulder and on the ipsilateral knee. As to the best of our knowledge, no paramagnetic disposable EMG electrodes are commercially available, we used the MR-ECG-electrodes and twirled a paramagnetic cable wire around the electrode button, as previously done in Marquetand et al. (2021).

During the measurement, the left ankle was pressed against the chair by a second person in the magnetically shielded room, and the participants were asked to push against it with the strongest, most continuous force possible. Three isometric contractions of the rectus femoris muscle were performed by the participants in 60 s blocks, with 30 s rest between each block (Figure 1A). Since the utilized OPMs could record only two orthogonal components of the magnetic field vector at a time (*y* and *z*, OPM sensor coordinates, Figure 1C), we repeated the measurements after rotating the sensors by 90° around their z-axis to record the magnetic field in all three spatial directions (X, Y, and Z, leg coordinates, Figures 1B,D). For this reason, the Z direction was recorded twice; therefore, we will refer to Z_X_ and Z_Y_ to indicate the Z component orthogonal, respectively, to the X and Y directions. To avoid unbalanced fatigue effects, the starting orientation of the OPM sensors was alternated across the subjects. Following the same logic, we labeled the EMG recordings according to the OPMs orientation, thus respecting the same alternating pattern. For clarity, from now on whenever we refer to OPM vector components or spatial directions, we refer to the leg coordinates system.

### Data Acquisition

MEG system (CTF Omega 275, Coquitlam, BC, Canada) electronics were used to record the analog output signal of the OPMs. The EEG channels of the same system were used to record the signals from the sEMG. Both OPM and EMG recordings were acquired with a sampling rate of 2343.8 Hz. The employed OPMs provide a magnetic field sensitivity in the order of 15 fT/$\sqrt{Hz}$ in a bandwidth of 3–135 Hz, an operating range below 200 nT, and a dynamic range of a few nanoteslas. To adapt to a non-zero magnetic background field, the sensors are equipped with internal compensation coils (Osborne et al., 2018) that can cancel magnetic background fields of up to 200 nT (operating range). For all measurements, the internal output gain factor of 3 was applied in the user interface of the sensors, which corresponds to a conversion factor for the analog output of the OPMs to magnetic flux of 1,11 nT/V.

### Data Analysis

Data analysis was performed in MATLAB (Version R2018b, MathWorks Inc., Natick, Massachusetts United States) using custom-built scripts and the open-source FieldTrip toolbox (Oostenveld et al., 2010). Continuously recorded EMG and OPM data were segmented in 3 trials of 60 s length, accordingly with the 3 isometric contraction sessions. In all the trials, 1 s of data-padding remained at the beginning and end of each trial to avoid edge artifacts due to filtering processes. The signals were demeaned and filtered using a 10 Hz high-pass, zero-phase, sixth order Butterworth infinite impulse response (IIR) filter. To suppress the powerline noise, a band-stop (frequency ranges 48–52; 98–102; 148–152), zero-phase fourth order Butterworth IIR filter was applied. Time-frequency analysis was performed using Morlet wavelet spectral power decomposition, with a Gaussian width of 15 (number of cycles) to maximize the frequency resolution. Time window was set to 100 ms, and the frequencies of interest ranged from 20 to 90 Hz. The magnitude of the signal was computed taking the square root of the power and averaged across the subjects. For the comparison with sEMG, the orthogonal vector components Y−Z and X−Z at each OPMs were summed by applying the Pythagorean theorem so that as:


$$a{\left(f\right)}_{xzi}=\sqrt{a{\left(f\right)}_{xi}^{2}+a{\left(f\right)}_{zi}^{2}}\;\;a{\left(f\right)}_{yzi}=\sqrt{a{\left(f\right)}_{yi}^{2}+a{\left(f\right)}_{zi}^{2}}\;$$

where a represents the magnitude of the frequency f; *x*, *y*, and *z* are the 3 different directions for the $i^{\mathrm{th}}$ OPM sensor. Accordingly, $a{\left(f\right)}_{xzi}$ represents the resultant magnitude of directions X and Z_X_; $a{\left(f\right)}_{yzi}$ represents the resultant magnitude of directions Y and Z_Y_. The computed resultant components were further averaged across subjects.

To quantify the spectral compression of the EMG and MMG signals, the spectral center of gravity (Cg) was used. The spectral center of gravity, which has also been defined mean frequency, centroid, or central frequency (Farina and Merletti, 2000; Georgakis et al., 2003; Du and Vuskovic, 2004; Oskoei and Hu, 2008; Phinyomark et al., 2012), represents here the weighted average of the frequency, calculated as the sum of product of the EMG/MMG spectrum magnitude and the frequency divided by the total sum of the spectrum magnitude. The definition of Cg is given by:


$$Cg=\sum_{i=1}^{M}f_{i}a_{i}/\sum_{i=1}^{M}a_{i}$$

where f indicates the $i^{\mathrm{th}}$ frequency bin, $a_{i}$ its respective magnitude, and M is the length of the frequency bins. The time-frequency decomposition of the signal was done using a sliding time window of 100 ms length. The Cg was calculated for each time window to quantify the shift of the spectral magnitude over time.

Finally, an individual estimation of the average signal strength was computed. The magnitude of the 4 vector components (separately considering Z_X_ and Z_Y_) was averaged for each OPM sensor to estimate the mean strength of the signal at different sensor position regardless of the component. In the same way, the signal magnitude of each vector component was averaged across the 4 OPMs to obtain a mean estimation of the strength of each component independently from the sensor position.

### Statistics

To test the hypothesis of a shift over time of the frequency spectrum as an indicator of muscle fatigue, the relation between time and Cg, in both sEMG and OPM signals, was estimated. After checking Cg values for normality using the Kolmogorov-Smirnov test, correlation (Pearson, two tailed) between Cg and time was calculated. A least-square regression line was fitted to the Cg data points to derive intercept and slope value as fatigue indices (Merletti et al., 1990; Farina and Merletti, 2000; Georgakis et al., 2003). Slope values of all subjects for sEMG and OPMs were further correlated (Pearson, one tailed) to quantify the similarity of the results between the two techniques. Finally, a comparison of all the individual averages signal magnitude, recorded at different sensors position and for different vector components, was performed. Repeated-measures ANOVA were used to assess the effect of sensors (within-subject factor, four levels: S1, S2, S3, and S4) and components (within-subject factor, four levels: X, Z_X_, Y, and Z_Y_) related to the signal strength. Greenhouse–Geisser correction was applied to adjust for violation of sphericity assumption. Two-tailed, paired sample t-tests were used to post-hoc compare the results.

## Results

Following the preprocessing, data were visually inspected. Five of the original twelve subjects had to be discharged due to a significant data loss caused by the partial detachment of the EMG reference electrode. The detachment was characterized by a clear flat line in the electromyographic signal which did not allow any further analysis. Hence, seven subjects were finally included in the analysis.

### Time-Frequency Analysis and Spectral Center of Gravity in OPM-MMG

**Figure 2:**
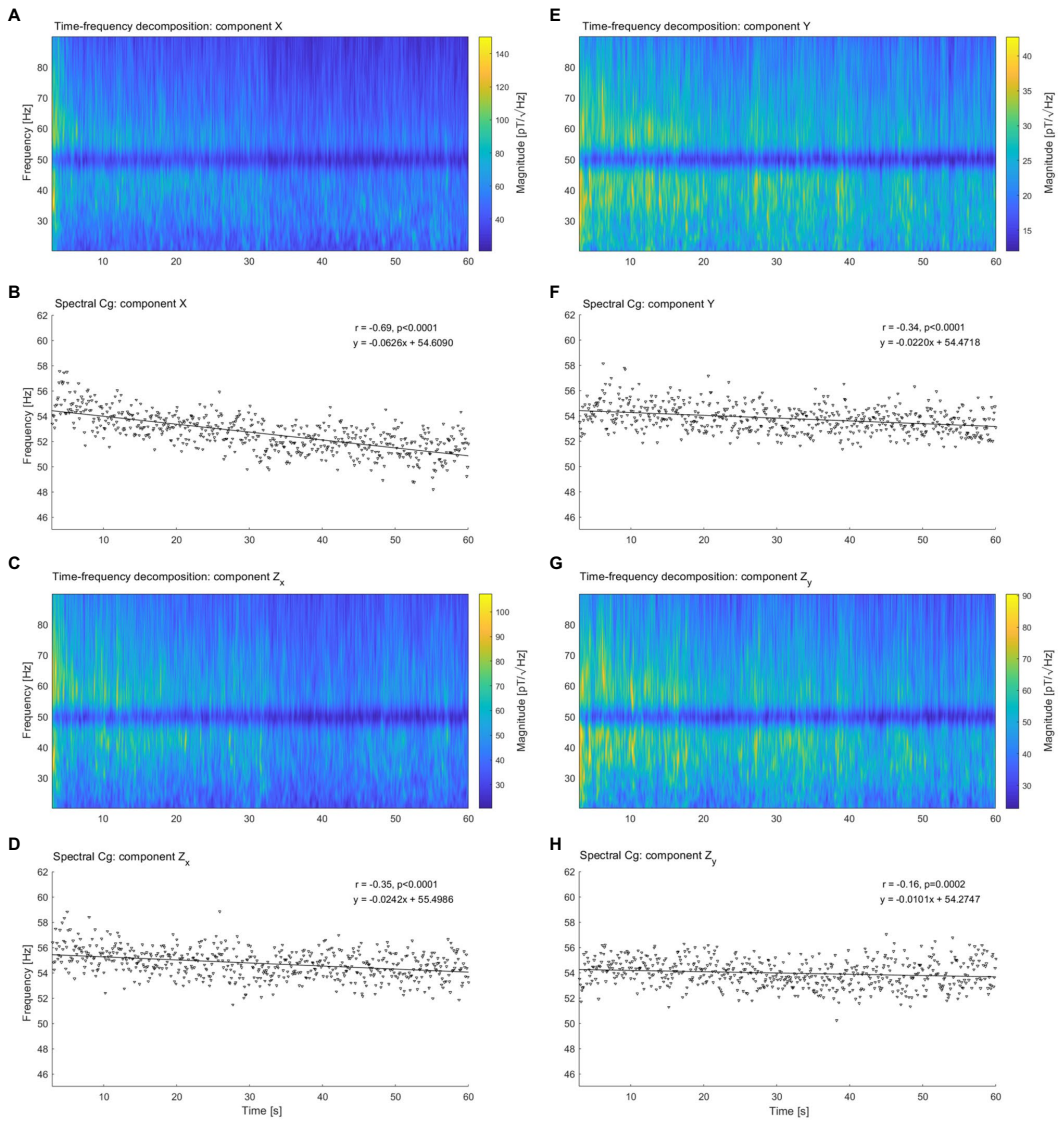
Spectral decomposition and spectral center of gravity of Optically Pumped Magnetometer Magnetomyography (OPM-MMG) vector components. **(A)** Spectral decomposition of component X, **(C)** Component Z_X_, **(E)** Component Y, and **(G)** Component Z_Y_. Time on the x-axis from 0 to 60 s, frequency range on the y axis from 20 to 90 Hz. Color bar depicting frequency magnitude. Scaling not match between the figure for visualization purposes, note that frequency magnitude of component X is one order of magnitude higher than component Y. Line noise signal suppression visible at 50 Hz. **(B)** Spectral center of gravity of component X, **(D)** Component Z_X_, **(F)** Component Y, and **(H)** Component Z_Y_. Each triangle represents a spectral center of gravity calculate for one of the 100 ms sliding time window utilized in the time-frequency analysis. Pearson’s r, point of intercept, and slope of the regression line quantified the spectral shift of the magnetomyographic signal over time.

Spectral decomposition was performed on each component (X, Y, and Z directions) of the magnetic field vector. In all three spatial directions, the spectral shift of the signal magnitude toward lower frequencies was already visible in the time-frequency analysis (Figures 2A,C,E,G). Correlation (Pearson, two tailed) between spectral Cg and time highlights a negative relationship in all the spatial direction. However, differences between the vector components were appreciable, with the X direction displaying a stronger decay (Figure 2B, r = −0.69, *p* < 0.0001, slope = −0.0626 Hz/s, intercept = 54.6090 Hz) of the spectral Cg over time, compared with the Y direction (Figure 2F, r = −0.34, *p* < 0.0001, slope = −0.0220 Hz/s, intercept = 54.4718 Hz). Interestingly, the same pattern was founded also for the Z components, where the Z_X_ component displayed a stronger decay (Figure 2D, r = −0.35, *p* < 0.0001, slope = −0.0242 Hz/s, intercept = 55.4986 Hz) compared to the Z_Y_ component (Figure 2H, r = −0.16, *p* = 0.0002, slope = −0.0101 Hz/s, intercept = 54.2747 Hz).

### Comparison between sEMG and OPM-MMG

**Figure 3:**
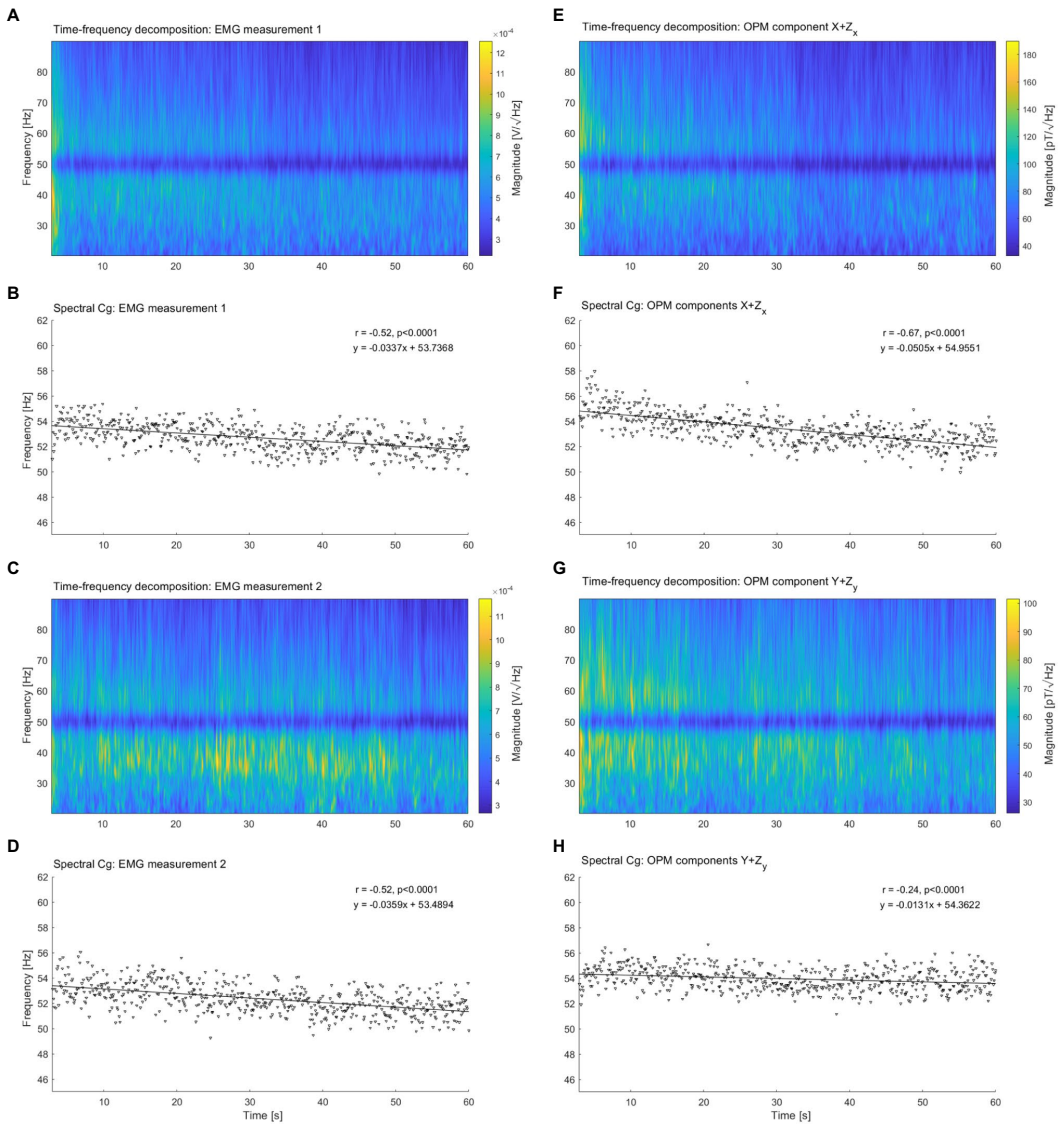
Spectral decomposition and spectral center of gravity of surface electromyography (sEMG) and OPM-MMG summed components. **(A,C)** Spectral decomposition of the two electromyography (EMG) measurement. For the comparison, OPM orthogonal components X−Z_X_ and Y−Z_Y_ were summed using Pythagorean theorem. Spectral decomposition has been performed on the resultant components X + Z_X_ **(E)** and Y + Z_Y_ **(G)**. Line noise signal suppression visible at 50 Hz. **(B,D)** Decrease of the spectral center of gravity over time of the EMG signals. **(F,H)** Decrease of the spectral center of gravity over time for the OPM components. Pearson’s r, point of intercept, and slope of the regression line quantified the spectral shift of the magnetomyographic signal over time.

After combing the orthogonal components of the magnetic field vectors X−Z_X_ and Y−Z_Y_ by applying the Pythagorean theorem, the computed resultant components named here X + Z_X_ and Y + Z_Y_ were compared with the sEMG signals. Again the spectral shift was visible in the time-frequency analysis of the 2 signals (Figures 3A,C,E,G), and confirmed by the analysis of the spectral Cg. However, while the spectral compression depicted by the sEMG was similar between measurement 1 (Figure 3B, r = −0.52, *p* < 0.0001, slope = −0.0337 Hz/s, intercept = 53.7368 Hz) and measurement 2 (Figure 3D, r = −0.53, *p* < 0.0001, slope = −0.0359 Hz/s, intercept = 53.4894 Hz), OPM-MMG spectral compression varies between components: X + Z_X_ (Figure 3F, r = −0.67, *p* < 0.0001, slope = −0.0505 Hz/s, intercept = 54.9551 Hz); Y + Z_Y_ (Figure 3H, r = − 0.24, *p* < 0.0001, slope = −0.0131 Hz/s, intercept = 54.3622 Hz).

**Figure 4:**
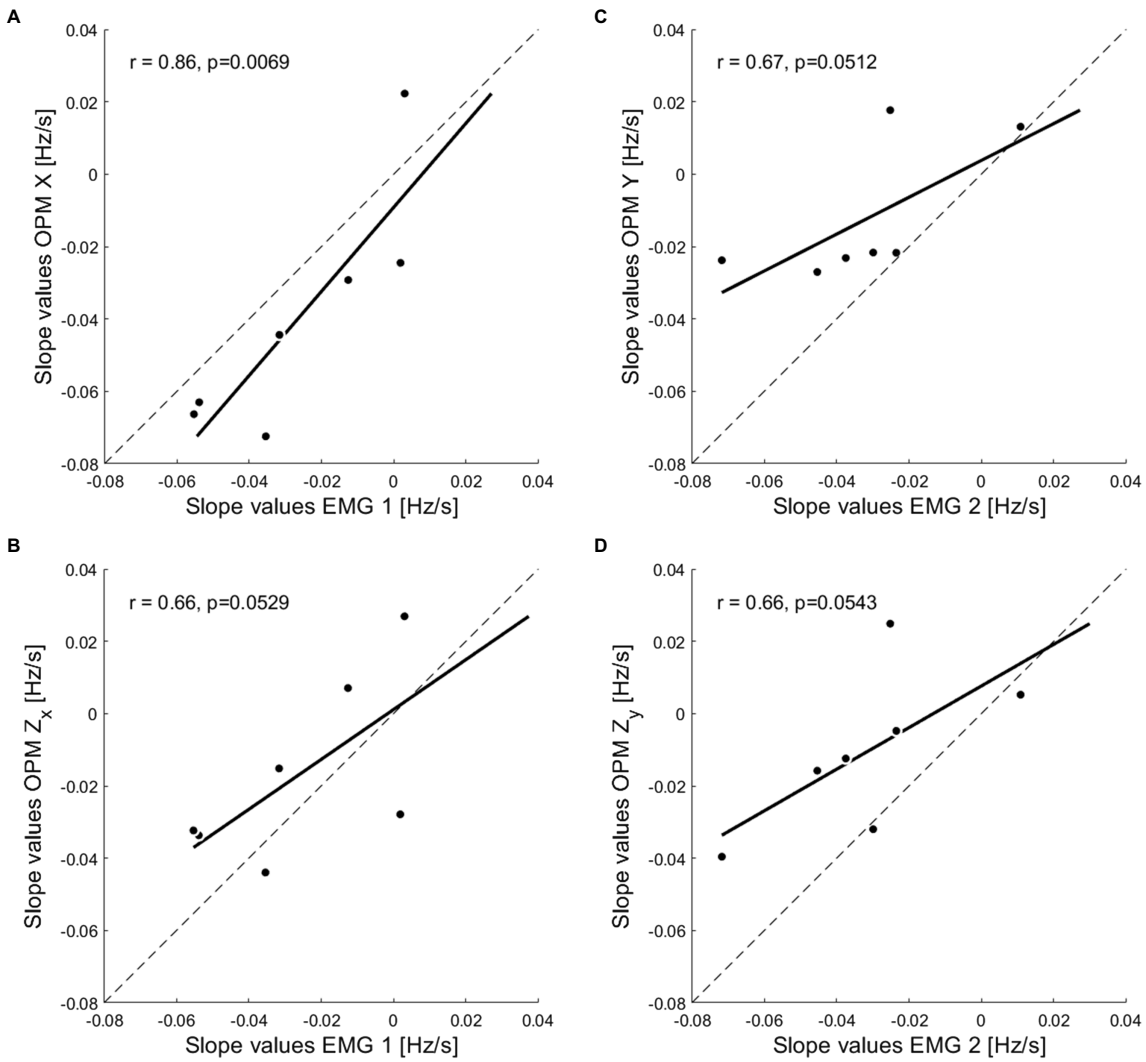
Correlation between OPM-MMG and sEMG single subject’ slope values. Pearson’s r coefficients obtained by correlating the single subjects’ slope values of each OPM vector components, with the slope values of the corresponding EMG measurement. **(A,B)** Correlations of the X and the orthogonal Z_X_ components with EMG measurement 1. **(C,D)** Correlations of the Y and the orthogonal Z_Y_ components with EMG measurement 2.

To quantify the similarity of the results between the two techniques, OPM slope values of each subject, for each component (Supplementary Material), were correlated (Pearson, one tailed) with the slope values of the respective EMG measurement session (Figure 4). The values reported for the X component revealed a high degree of similarity with the corresponding EMG values (Figure 4A, r = 0.86, *p* = 0.0069), while for all the other components only a tendency toward a positive correlation was found (Z_X_ Figure 4B, r = 0.66, *p* = 0.0529; Y Figure 4C, r = 0.67, *p* = 0.0512; and Z_Y_ Figure 4D, r = 0.66, *p* = 0.0543).

### Comparison of the Signal Strength Between OPM Sensors Position and Vector Components

**Figure 5:**
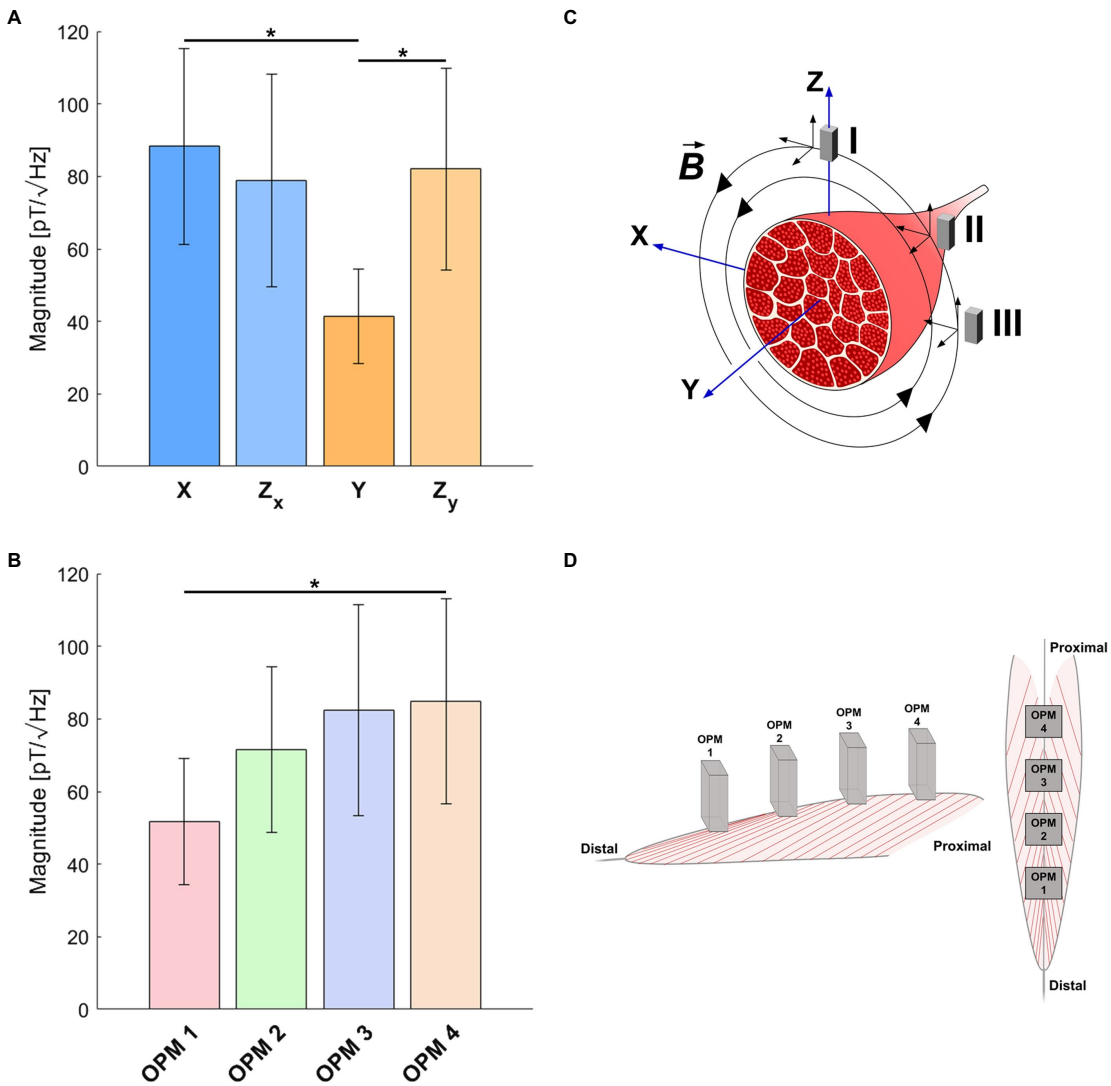
Comparison of the average signal strength at different OPM sensors position and for different vector components. **(A)** Averaged signal strength across the 4 OPM sensors is compared between vector components. **(B)** Averaged signal strength across the 4 vector components (Z_X_ and Z_Y_ separately considered) is compared between the 4 OPMs. Error bars indicate SEM across participants (^*^p<0.05 ). **(C)** Model of the magnetic field generated by the electric activity of the muscle. Signal strengths of the recorded spatial components of the magnetic fields are a function of the propagation direction of the motor action potential along the muscle fibers and the sensor positions and orientations. For a detailed explanation of the model and the speculations that can be drawn from the strength of the magnetic field components for the propagation direction of muscular electrical activity, refer to the discussion. **(D)** Graphic representation of the OPMs distribution over the muscle.

Considering the differences between vector components highlighted by the previous results, an analysis of the average signal strength measured at different sensors position for different components was performed. ANOVA on signal magnitude revealed a significant main effect of both sensors position *F*(3,18, *ε* = 0.471) = 6.241, *p* = 0.027 and vector components *F*(3,18, *ε* = 0.598) = 4.7, *p* = 0.037. No significant sensors position x components interaction was found *F*(9,54, *ε* = 0.164) = 1.317, *p* = 0.303. Post-hoc comparisons showed significant differences in signal magnitude between both component X (*p* = 0.021, two-tailed paired *t*-test) and Z_Y_ (*p* = 0.036, two-tailed paired *t*-test) compared to component Y (Figure 5A, and between sensor 1 and 4 *p* = 0.027, two-tailed paired *t*-test; Figure 5B).

## Discussion

In this first magnetomyographic investigation of muscle fatigue with OPM sensors, we showed a spectral compression of the MMG signal, highlighted by the decrease of the spectral center of gravity over time. This compression was comparable with the one of the electromyographic signal that was simultaneously recorded through sEMG, and which is regarded as a distinctive marker of muscle fatigue (Kogi and Hakamada, 1962; Viitasalo and Komi, 1977; Petrofsky et al., 1982; De Luca, 1984; Duchêne and Goubel, 1990; Doud and Walsh, 1995; Lowery et al., 2000; Beck et al., 2014).

Interestingly, while for the EMG signal, the spectral compression was closely matched between measurements, MMG signal compression varied considerably depending on the different recorded components of the magnetic field vector (refer to leg coordinates system Figures 1B,D). The clearest spectral compression was found for the X component. This finding was also supported by a significant correlation between the single subjects’ slope values for the X component, with the values of the corresponding EMG measurement, which highlight the high similarity of the results reported by the two techniques. For all the other vector components (Z_X_, Y, and Z_Y_), the picture is more blurred. Even though all the different signals displayed a significant spectral compression over time, the entity of this compression was lower compared to the one showed by the X component. Moreover, the correlation of the single subjects’ slope values with the corresponding EMG measurement reported only a tendency rather than a significant positive correlation. This was true not only for the components Y and Z_Y_ which were recorded on a different measurement after the rotation of the sensors, but also for the Z_X_ component that is orthogonal to X. However, since the sample size sensibly influences the significance of the results even in presence of a strong correlation, the latter finding can be explained by the small number of subjects included in the experiment and can therefore be regarded as a limitation of the study. Yet, it is evident that different components of the MMG signal relate differentially to the EMG signal, enabling speculations on the major direction of the magnetic field.

As depicted in the model shown in Figure 5C, the strengths of the recorded vector components vary as a function of the position and orientation of the OPM sensors. Assuming a motor action potential traveling along the Y axis, a circular magnetic field in the X−Z plane is generated. In this situation, a sensor placed in position I would record only the X component of the magnetic field vector, a sensor placed in position II would capture a combination of the X and Z components, while a sensor placed in position III would measure exclusively the Z component. Since the magnetic field vector in the Y direction would be zero, no magnetic activity could be recorded in this direction regardless the orientation of the sensors. In our data (Figure 5A), the lowest signal strength was observed for the Y component, while components X and Z were similar. By comparing this finding with the model, we can speculate that the propagation of the MAPs occurs mainly along the Y axis. Considering the comparable signal strength of the X and Z components, we can reasonably exclude the possibilities described by position I and III where a low signal would be expected in two of the recorded components (Z and Y for position I; X and Y for position III). Hence, a situation like the one depicted by position II seems to be the one that better fit our data, in which the only one component displaying a low signal correspond to the propagation direction of the muscular action potentials. The interpretation of this result must be made cautiously since we can only speculate on the propagation direction of the MAPs, by looking at the major direction of the magnetic field. Any spatial information regarding the propagation of the signal at the source level requires the implementation of source localization algorithms that involve volume conduction models which are currently not yet developed for the rectus femoris muscle. Also, the comparison of the signal magnitude recorded at different OPM sensors (Figure 5B), highlighted a progressive increment of the strength of the signal between the sensors, with a significant difference between sensor 1 and 4. The understanding of this finding needs further investigations. One possible explanation might be related to the structure of the rectus femoris. The muscle originates from the anterior inferior iliac spine and the portion of the ilium above the acetabulum and develops with a fusiform shape until the insertion into the base of the patella. Due to its fusiform shape, the superficial surface of the rectus femoris increases from distal to proximal to decrease again at the origin. Considering the arrangement of the sensors in our study (Figure 5D), sensor 1 was in a distal position compared to sensor 4, and therefore, the higher signal magnitude recorded in the latter could reflect the overall muscle surface size. Another explanation for this finding, however, is the varying distance of the sensors from the source. Depending on the sensor position, the distance of the OPM from the skin surface, hence from the muscle, changed during the isometric contraction. Because of the decay of the magnetic field strength proportional to 1/r^3^, where r represents the distance from the source, the magnitude of the signal increases with the proximity to the source. Therefore, the lower signal strength recorded at sensor 1 might also reflect the larger distance of the sensor from the source, which decrease from distal to proximal resulting in a significant higher signal strength at sensor 4. Even though this was beyond the scope of this study, further investigations need to account for this factor. A possible solution would be to integrate a light distance detector to each sensor to real-time monitor the position of each OPM with respect to the skin surface.

Finally, some considerations regarding the potential of OPM-MMG to become a valid alternative to sEMG. Typically, sEMG used disposable silver/silver chloride (Ag/AgCl) electrodes which require a careful skin preparation (Hermens et al., 1999), and the application of a conductive gel to reduce the electrode-skin impedance and enhance conductivity. From a clinical perspective, this process is time consuming, it might be unpleasant for the patients since it requires a mild skin abrasion (Tam and Webster, 1977). And in some rare cases may even lead to adverse dermatological responses to electrolyte gels (Searle and Kirkup, 2000). The presence of skin rashes, scars, scabs, or areas with lesioned skin (e.g., burns or cuts) pose additional limitations to the use of sEMG. Furthermore, intrinsic noise sources, such as electro-chemical noise, originating at the electrode-skin interface may contaminate the sEMG signal (Huigen et al., 2002; De Luca et al., 2010). For all these limitations, contactless OPM-MMG measurement can offer an advantage. Yet, OPM technology is currently under development; therefore, the high cost and the requirement of magnetic shielding still limit its use to research context. However, in view of the promising advancement of wearable OPM systems for MEG application (Boto et al., 2019; Roberts et al., 2019; Boto et al., 2021; Seymour et al., 2021), and the progress in development of OPM-optimized magnetic shielding solutions (Holmes et al., 2018; Iivanainen et al., 2019; Borna et al., 2020; Zhang et al., 2020), it is foreseeable a broader diffusion of the technology in the future with a consequential decrease of the costs. This would paved the way toward the development of wearable OPM-MMG systems (Zuo et al., 2020), for the measurement and assessment of muscle activity for research and diagnostic purposes.

## Conclusion

It can be summarized that is possible to detect muscle fatigue through magnetomyography with OPMs. OPM-MMG and sEMG slope values, which quantified the spectral compression of the signals over time as index of fatigue, were positively correlated exhibiting similarity between the techniques. Additionally, the capability of OPMs to record different components of the magnetic field vector enables speculations regarding the propagation direction of the MAPs during isometric contraction. Yet, these speculations need to be verified through the implementation of source reconstruction algorithms, which account for sensors position, muscle geometry, and volume conduction properties, allowing to estimate muscle activity at a source level. Overall, this first magnetomyographic investigation of muscular fatigue suggests that OPM-based MMG offers a promising new approach of study in muscle physiology.

## Data Availability Statement

The data supporting the findings of this study are available on request to the corresponding author.

## Ethics Statement

The study was performed in accordance to the Declaration of Helsinki (World Medical Association, 2001). The participants are also authors of this publication and gave informed consent for their data to be published.

## Author Contributions

JM and DSo: study design, data collection, data analysis, writing and revision. LS: writing and revision. SB: data analysis and revision. HC, GR, CK, MK, AR, JH, DSt, and JO: data collection and revision. TM: instrumentation, setup and revision. JD: setup, study design and data collection. CB and PB: data analysis and revision. All authors contributed to the article and approved the submitted version.

## Conflict of Interest

The authors declare that the research was conducted in the absence of any commercial or financial relationships that could be construed as a potential conflict of interest.

## Publisher’s Note

All claims expressed in this article are solely those of the authors and do not necessarily represent those of their affiliated organizations, or those of the publisher, the editors and the reviewers. Any product that may be evaluated in this article, or claim that may be made by its manufacturer, is not guaranteed or endorsed by the publisher.

## Supplementary Material

**Supplementary Material 1** | Single subjects’ spectral center of gravity, Pearson’s r, slope values, and point of intercept for the X and Z_X_ components.

**Supplementary Material 2** | Single subjects’ spectral center of gravity, Pearson’s r, slope values, and point of intercept for the Y and Z_Y_ components.

**Supplementary Material 3** | Single subjects’ spectral center of gravity, Pearson’s r, slope values, and point of intercept for the two EMG measurements.

## Abbreviations

OPM: Optically Pumped Magnetometer
EMG: Electromyography
sEMG: Surface Electromyography
MMG: Magnetomyography
OPM-MMG: Optically Pumped Magnetometer Magnetomyography
MEG: Magnetoencephalography
MAP: Muscular Action Potential
