# Supplementary figures and images for: Muscle Fatigue Revisited – Insights From Optically Pumped Magnetometers
Source: Front Physiol. 2021 Dec 17;12:724755. doi: 10.3389/fphys.2021.724755 (PMC8718712; doi:10.3389/fphys.2021.724755)

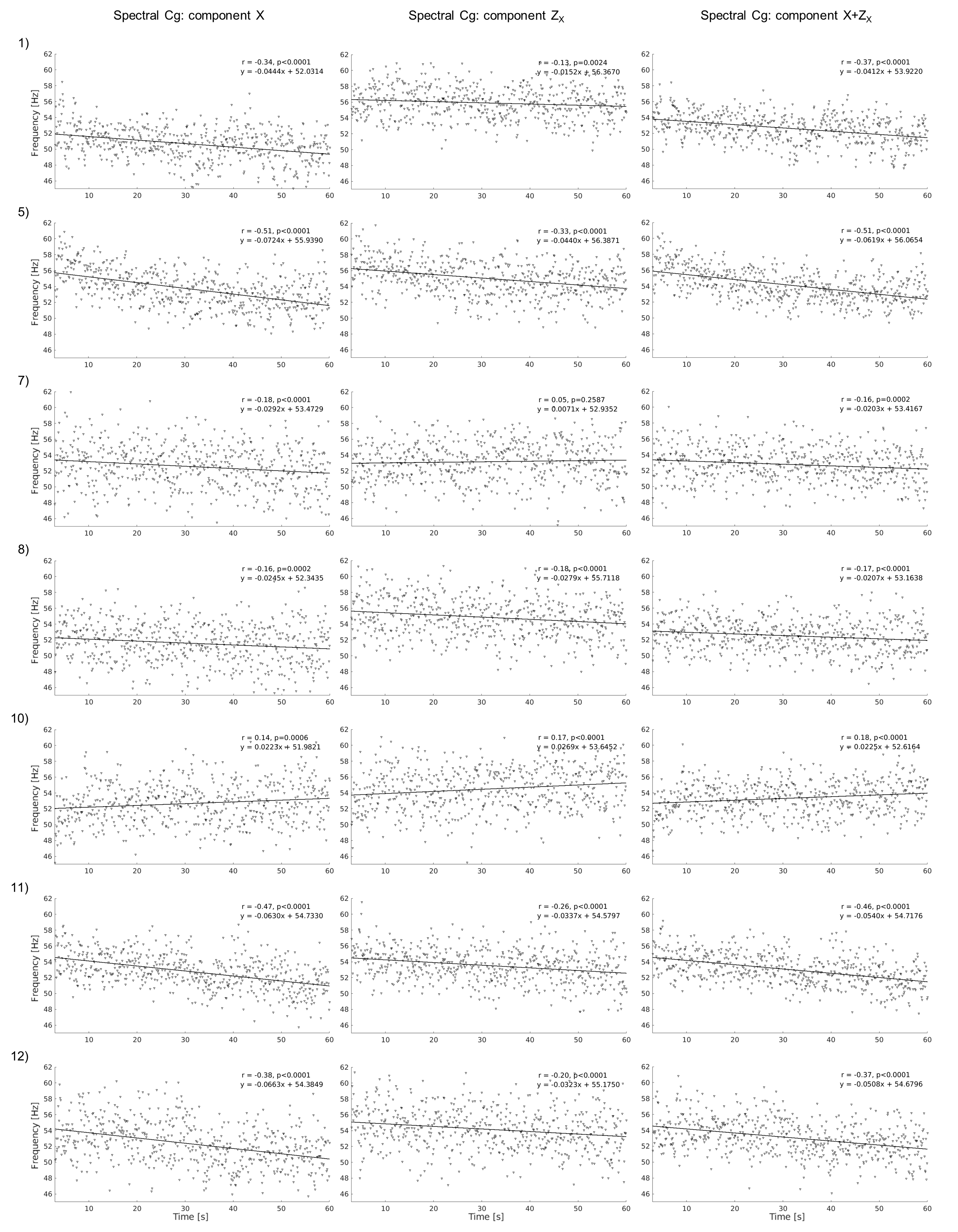

Supplement: Supplementary file 1 — Supplementary Material 1 | Single subjects’ spectral center of gravity, Pearson’s r, slope values, and point of intercept for the X and ZX components. [file Image_1.TIF]

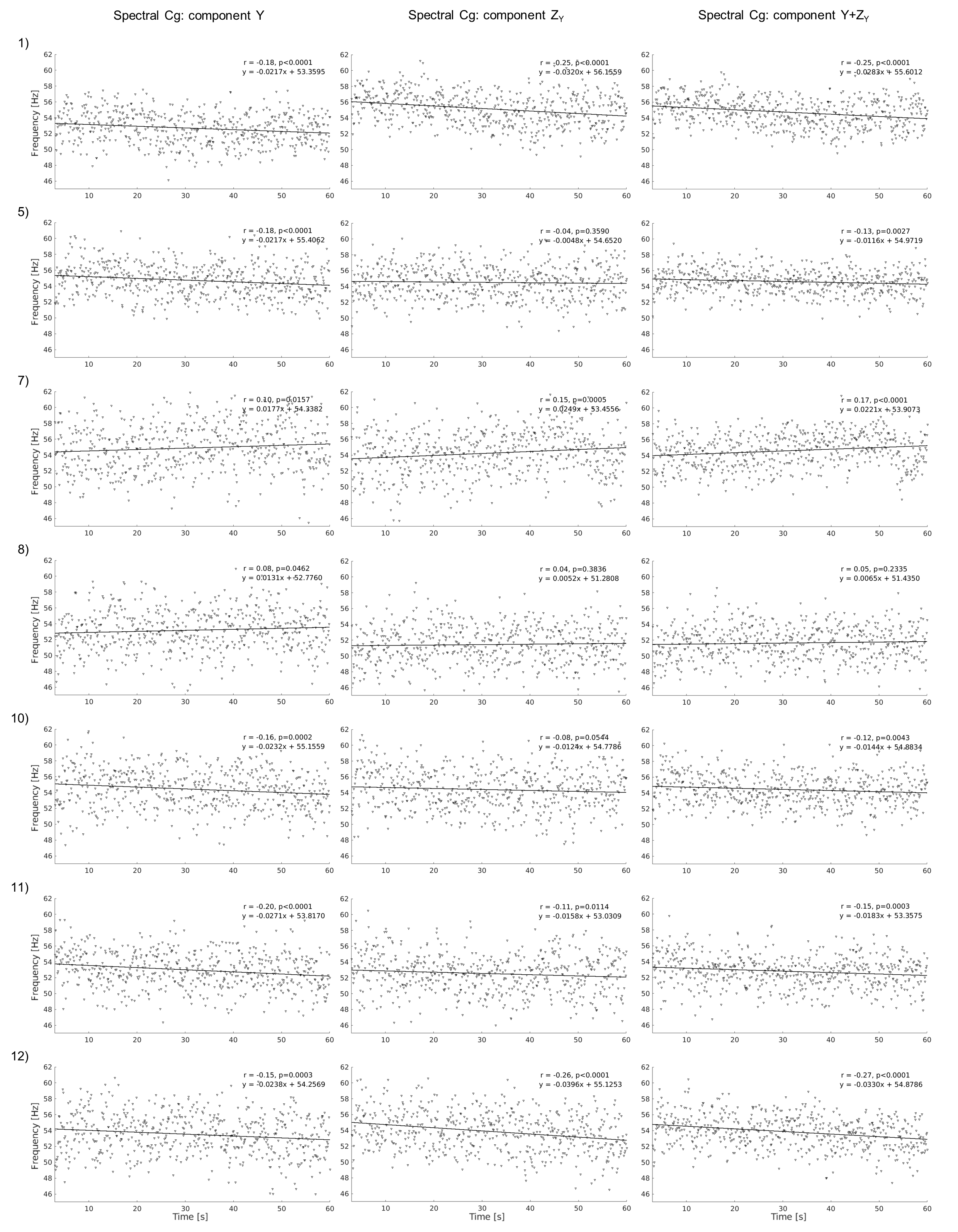

Supplement: Supplementary file 2 — Supplementary Material 2 | Single subjects’ spectral center of gravity, Pearson’s r, slope values, and point of intercept for the Y and ZY components. [file Image_2.TIF]

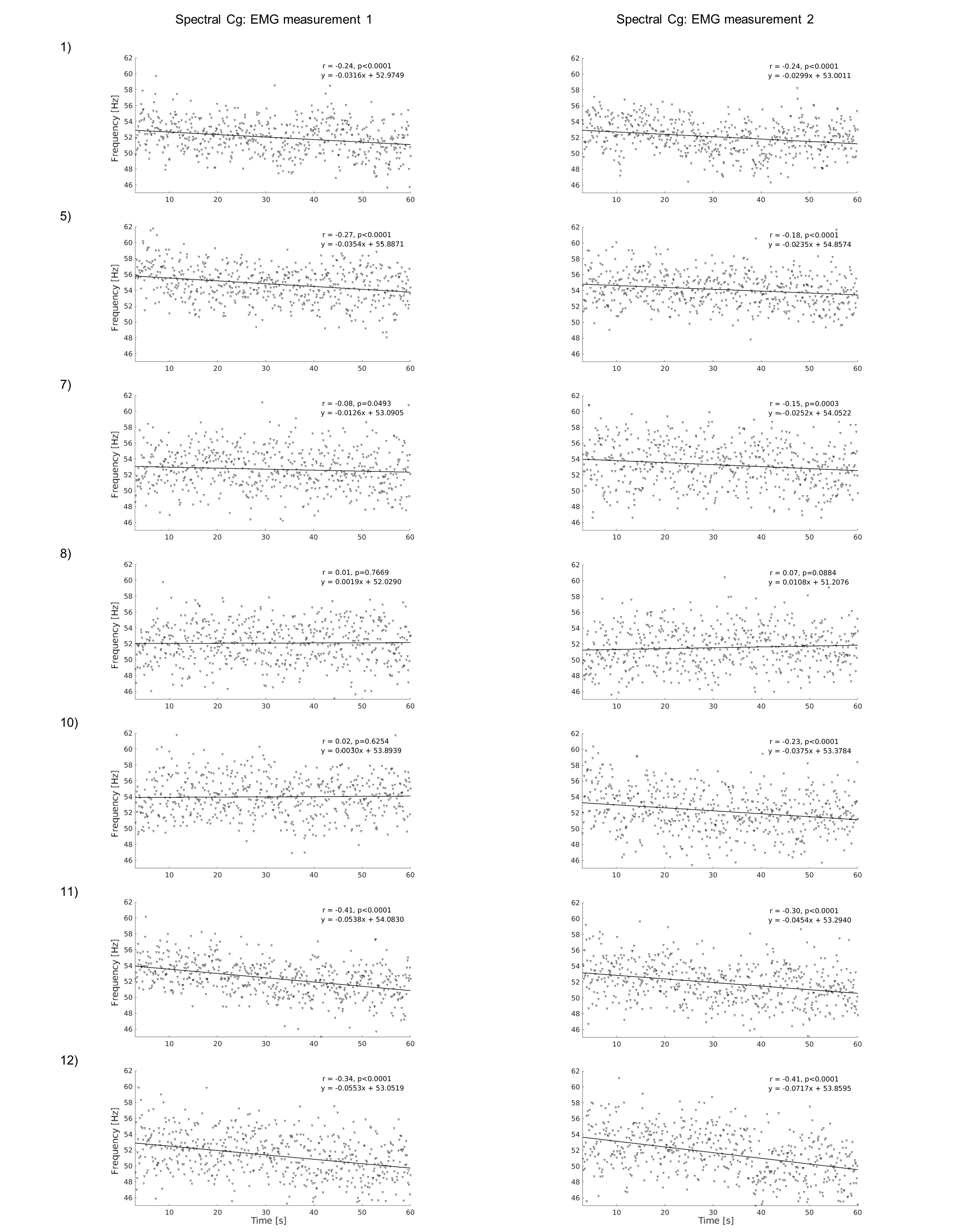

Supplement: Supplementary file 3 — Supplementary Material 3 | Single subjects’ spectral center of gravity, Pearson’s r, slope values, and point of intercept for the two EMG measurements. [file Image_3.TIF]
